# Plantar nerve neural fibrolipoma: rare neuroma arising from the plantar nerve of the heel

**DOI:** 10.1093/jscr/rjac361

**Published:** 2022-09-19

**Authors:** Raghav Nand, Dakshinamurthy Sunderamoorthy

**Affiliations:** Trauma and Orthopedics, Scunthorpe General Hospital, Scunthorpe, UK; Trauma and Orthopedics, Scunthorpe General Hospital, Scunthorpe, UK

**Keywords:** plantar nerve, neural fibrolopoma, neuroma

## Abstract

Every year, as many as 2 million patients worldwide present with plantar heel pain, with men and women being affected equally. This case reports reflects upon a healthy 46-year-old female who presented with a subtype of true neoplasm called as neural fibrolipoma arising from the plantar nerve. This 17 × 7 × 11-mm painful soft tissue mass was firstly detected by USS, which showed this mass extended deep to the plantar fascia and not arising from it, hence ruling out the common misconception of plantar fibromatosis. Later, as discussed in the Yorkshire Sarcoma MDT with magnetic resonance imaging images, a malignancy was ruled out. The patient eventually opted for an excision biopsy which confirmed the neural fibrolipoma followed by an excellent outcome at a 2-year review. This case report highlights the need to consider the neural fibroplipoma as a rare case of plantar/heel pain, and excision of the lesion would provide an excellent outcome.

## INTRODUCTION

A neuroma can be defined as benign tumour of the nerve tissue. Neuromas can be differentiated into three categories. Firstly, as a true neuroma, and secondly, as neuromas associated with trauma, and finally, as neuromas as part of a syndrome such as neurofibromatosis or Multiple Endocrine Neoplasia (MEN2b). Neuromas are overlooked as a differential diagnosis for heel pain, and they are diagnosed by a combination of physical examination, history taking, radiological imaging and by histopathological findings following excision [1].

A neural fibrolipoma, also known as a lipofibromatous hamartoma, is a benign, soft-tissue neoplasm generated by the proliferation of mature nerve sheet fibroblast and adipocytes, leading to the formation of a palpable subcutaneous mass [2]. The exact pathogenesis of a neural fibrolipoma is unknown, however, chronic irritation and trauma have been known to play a precipitating factor. Often seen in nerves with high risk of compression, such as the median nerve, it is very rare to be present in the plantar nerve.

## CASE REPORT

A healthy 46-year-old female was referred by her General Practitioner for an evaluation of a painful soft tissue mass present in the right plantar aspect of the midfoot. The mass had been present for ~2–3 years and had recently increased in size in the last 4 months. The patient described the feeling as walking on a hard stone. There was no history of trauma or significant family history. Upon inspection, there was no apparent infection or skin changes. Upon examination, the patient had bilateral planovalgus feet with bilateral hallux valgus worse on the right side. This swelling had led to chronic pain with some sensitivity and numbness over that area. Following the patient’s first appointment, the patient received an injection of 80 mg depo-medrone and 1 ml of 1% lignocaine under ultrasound (US) guidance into the second metatarsophalangeal to temporarily provide symptomatic relief for the pain, while an US was arranged to rule out plantar fibromatosis. X-rays ruled out any bony injury or obvious bone malignancy.

**Figure 1:**
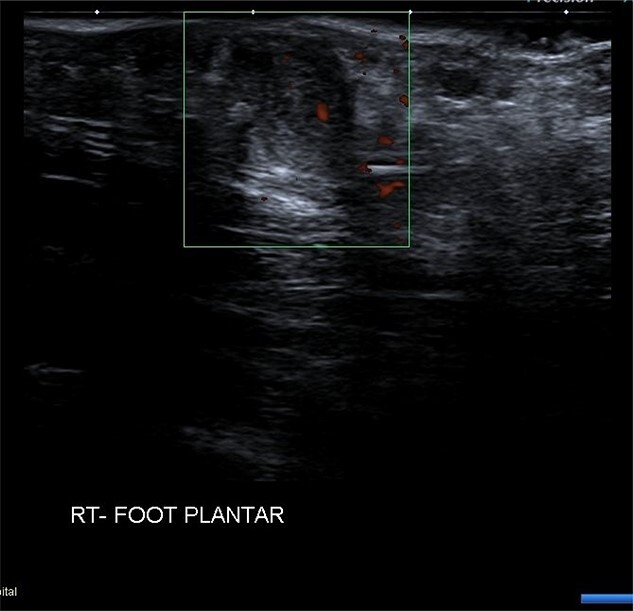
USS right foot plantar showing mass deep to plantar fascia.

**Figure 2:**
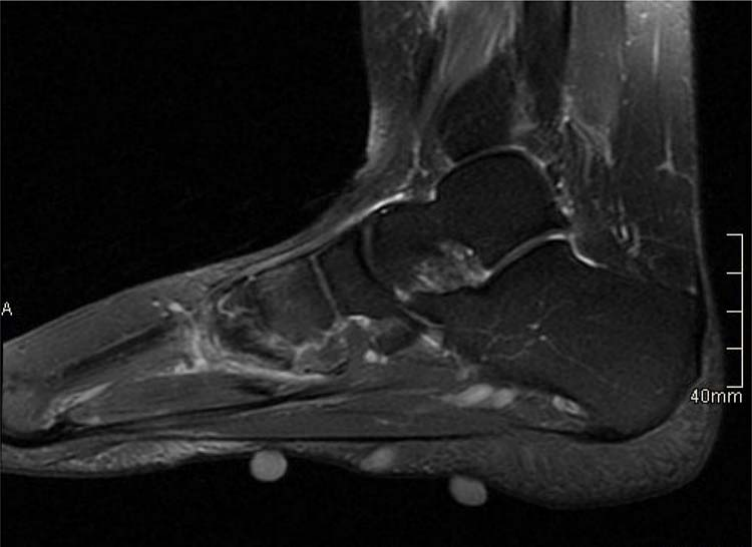
MRI with contrast; right foot—sagittal view.

**Figure 3:**
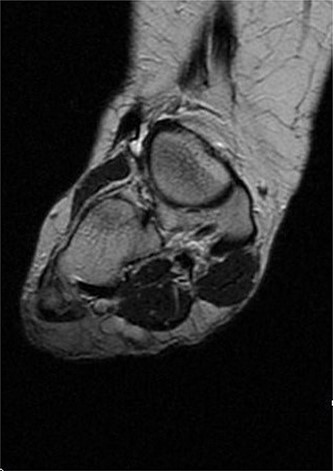
MRI with contrast; right foot—T2 coronal view.

USS described this swelling in the plantar aspect of the midfoot as a well-defined, hypoechoic tender lesion within the plantar subcutaneous tissue measuring ~17 × 7 × 11 mm. More importantly, the lesion extended deep to the plantar fascia not arising from it, hence ruling out plantar fibromatosis (Fig. 1). In order to rule out any aggressive pathology, a magnetic resonance imaging (MRI) with contrast was arranged, which revealed a 2 cm by 5 mm tubular oblique lesion in the plantar subcutaneous fat. MR imaging was also able to rule out sinus tarsi syndrome and any internal derangements (Figs 2 and 3). She was referred to Orthotics for a medial support arch to help with pain. Since the exact characteristics of this lesion were still unknown, the case was referred to the Yorkshire Sarcoma Multi-disciplinary team (MDT). The outcome of this meeting concluded that there were no radiological features of a sarcoma. She still experienced an excruciating, sharp stabbing pain in her right foot. Pain score was 10/10. She opted for the surgical excision biopsy of the lesion. The post-operative period was uneventful with good healing of the wound. She was followed up 2 months, and with the histology findings, results revealed a rare soft tissue neural neoplasm in keeping of a neurofibrolipoma. The patient was happy with the overall outcome and was discharged. She was reviewed again after 2 years for a further foot and ankle problem in the other foot, and at the time of review, she was completely pain-free in the right foot with an excellent outcome.

## DISCUSSION

A true neuroma can come in various sizes and locations, including a vestibular schwannoma, acoustic neuroma, ganglioneuroma, neurothekeoma and nerve sheath myxoma. These neuromas have their own unique characteristics and origin. The true incidence of many these neuromas vary in different literature, as often neuromas go undetected until they become symptomatic [3]. In the cases of an acoustic neuroma or vestibular schwannoma, patients can present with tinnitus hearing loss and vertigo. In this case, the patient presented with chronic pain in her right foot [4].

When reviewing such patients, it is imperative to rule out any malignancy before excising the mass, which is why referring this patient to a Sarcoma MDT was essential. Other infectious differentials, including septic arthritis and osteomyelitis, were ruled out early since blood counts showed C-reactive protein—1.2 mg/l, white cell count—5.1 10 ^*^ 9/l and neutrophils—3.47 10 ^*^ 9/l. Arthritic differentials, such as Gout, were also ruled out based on blood parameters which revealed urate—287. Given that the pain was focused on the plantar aspect of the heel and not primarily in the posterior heel, conditions such as Achilles tendonitis, retroachiles bursitis or retrocalcaneal bursistis were ruled out. Furthermore, given that the patient was not an adolescent, Severs disease (calcaneal apophysitis) was also excluded [5].

It is important to recognize that this true neuroma originated from a peripheral nerve, such as the medial plantar nerve, and not from the inter-digital nerve as that would make it a Mortons neuroma [6].

During excision, the medial plantar nerve was preserved, ensuring the patient preserved motor function of the abductor hallucis, flexor digitorum brevis, the flexor hallucis brevis and the first lumbrical muscle.

## CONCLUSION

This case report highlights the need to consider the neural fibroplipoma as a rare case of plantar/heel pain, and excision of the lesion would provide an excellent outcome.

## CONFLICT OF INTEREST STATEMENT

None declared.

## FUNDING

None
